# Metastatic Seeding From a Gastrointestinal Neoplasia in a Pituitary Adenoma: A Case Report and Literature Review

**DOI:** 10.7759/cureus.34676

**Published:** 2023-02-06

**Authors:** Charles Gariépy, Pierre-Olivier Champagne

**Affiliations:** 1 Medicine, Laval University, Quebec City, CAN; 2 Neurosurgery, Enfant-Jesus Hospital, Laval University, Quebec City, CAN

**Keywords:** endoscopic craniotomy, gastro oesophageal cancer, pituitary metastasis, composite tumor, prolactinoma

## Abstract

Pituitary composite tumors consisting of metastasis within an adenoma are rare and aggressive entities. We present a case of esophageal adenocarcinoma metastasis at a prolactinoma presenting in a unique fashion and highlight how this case could contribute to a better understanding and early recognition of this condition. The patient was a 65-year-old male who presented with partial palsy of the third and sixth cranial nerves. He had a history of treated esophageal adenocarcinoma. He also had a known small parasellar lesion, with an elevated prolactin. Investigations showed a rapid progression of the parasellar lesion and normalization of prolactin. Partial surgical resection was performed, and pathology confirmed metastasis of the known digestive tract neoplasia. Although extremely rare, dual pathology of pituitary metastasis within adenomas should be considered in the differential diagnosis of sellar masses. Atypical behavior of benign adenomas, including rapid growth, spontaneous normalization of prolactin, or progression despite medical treatment should prompt medical teams to reconsider their diagnosis.

## Introduction

Pituitary tumors are the most common intracranial neoplasm after meningioma [1]. The most common pituitary tumors are adenomas. A 2015 population study identified that 43.0% of pituitary adenomas were non-functional, while 39.9% were prolactinomas [2]. Pituitary metastasis is a rare entity, representing only 2% of all pituitary lesions [3]. Although rarely diagnosed, autopsy series show a high prevalence of pituitary metastasis: 27% of patients diagnosed with malignant cancer had pituitary metastasis [4]. Only 7% of patients would be symptomatic of these lesions, which explains why they are so rarely diagnosed. Breast and pulmonary cancers are the most common primary tumor to metastasize to the pituitary [5].

Composite tumors are neoplasias showing features of two distinct histologic entities. A lesser-known and extremely rare entity concerning the pituitary is a composite tumor consisting of metastasis within a pre-existing pituitary adenoma, with only a few cases reported in the literature [6]. In this case report, we present a case of a gastrointestinal (GI) tract neoplasm that metastasized into a prolactinoma and highlight its unique features as well as the importance of and potential signs for early recognition of this diagnosis.

## Case presentation

Patient information

A 65-year-old patient was referred to the neurosurgery department by his primary care physician for the onset of new diplopia. The patient had a previous history of benign prostatic hypertrophy and esophageal adenocarcinoma, surgically removed by an Ivor-Lewis type esophagectomy a year prior. The patient had a CROSS type neoadjuvant treatment consisting of radiotherapy and chemotherapy prior to surgery. The tumor was classified as a T2N2 esophageal adenocarcinoma with four out of 31 lymph nodes being positive for tumor. No further treatment was deemed necessary after surgery.

**Figure 1:**
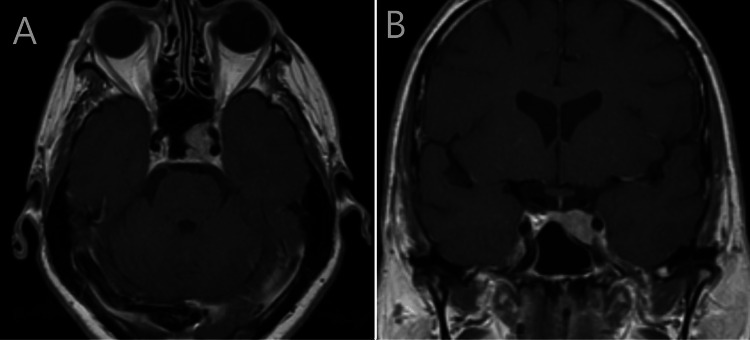
Initial imaging five years prior to visual symptoms Axial (A) and coronal (B) T1-weighted gadolinium-enhanced MRI showing the left parasellar lesion when it was initially discovered, five years before the patient became symptomatic. Imaging shows a homogenous enhancing lesion centered on the left cavernous sinus MRI: magnetic resonance imaging

The patient also had a known intracanalar vestibular schwannoma (Koos grade 1) that had been diagnosed via MRI in the context of severe hearing loss of the right ear five years prior. At that time, five years ago, a lesion centered on the left cavernous sinus had been incidentally discovered (Figure 1). Endocrinologic investigations are shown in Table 1. The lesion had been radiologically and biochemically diagnosed as a macroprolactinoma. Unfortunately, he had been lost to follow-up and no treatment had been offered at that time. He had not undergone any control MRI until he presented with his new-onset diplopia (present case).

**Table 1:** Endocrinologic investigations at first lesion discovery and at the time of consultation FSH: follicle-stimulating hormone; IGF-1: insulin-like growth factor 1; LH: luteinizing hormone; TSH: thyroid-stimulating hormone

Endocrinologic workup	Initial value	Value at symptomatic consultation
Prolactin (normal value: <16 μg/L)	496 μg/L	28 μg/L
Total testosterone (normal value: 8.3–33.0 nmol/L)	4.4 nmol/L	1.3 nmol/L
Cortisol	533 nmol/L (8h00)	207 nmol/L (14h00)
TSH (normal value: 0.5–5 mUI/L)	2.62 mUI/L	1.32 mUI/L
IGF-1 (normal value: 35–200 µg/L)	113 µg/L	102 µg/L
FSH (normal value: 2.0–15 UI/L)	-	1.9 UI/L
LH (normal value: 1.5–10 UI/L)	1.0 UI/L	0.7 UI/L

Presenting complaints and clinical findings

At presentation, neurological examination revealed a partial left third cranial nerve palsy and partial left sixth cranial nerve palsy. Visual fields were normal. The rest of the neurological exam was without particularity. The patient also reported a loss of libido and erectile dysfunction for the last five years. No increased diuresis was reported by the patient, nor any increased thirst.

Investigation and management

**Figure 2:**
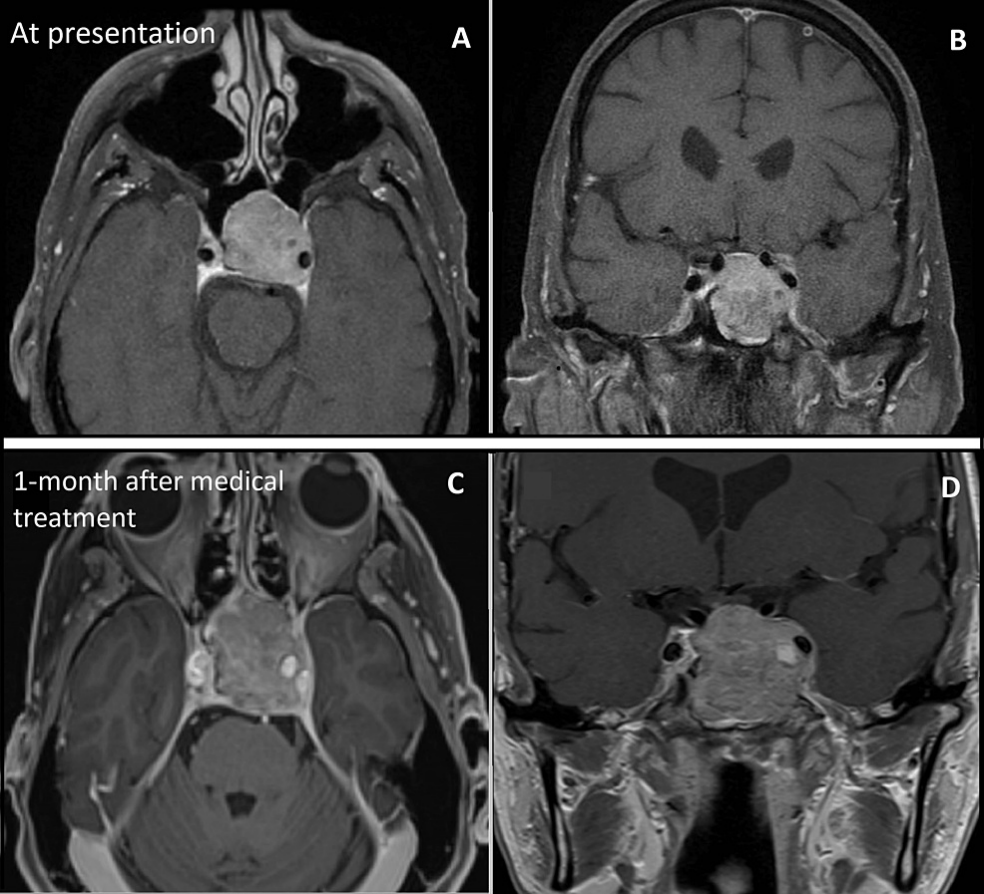
Imaging at the time of consultation and after one month of medical treatment A, B: Axial (A) and coronal (B) T1-weighted gadolinium-enhanced MRI showing the parasellar lesion when the patient consulted for his visual symptoms. The lesion substantially grew over the years. C, D: Axial (C) and coronal (D) MRI sequences show the abnormally rapid growth of the lesion one month after the medical treatment was started MRI: magnetic resonance imaging

MRI showed a significant progression of the left cavernous sinus lesion from the previous imaging five years ago, which now measured 30 mm x 31 mm x 29 mm (previously 15 mm x 18 mm x 16 mm) (Figure 2). An endocrinologic workup was ordered and prolactin was measured at 28 μg/L (Table 1). Since prolactin had been previously measured at 496 μmol/L, total testosterone was low, and given the fact the patient presented with clinical hypogonadism, the medical team concluded that the most likely diagnosis was still a macroprolactinoma, even though the spontaneous decrease in prolactin levels was unconventional. A trial of bromocriptine at 2.5 mg once daily was ordered with close clinical and radiological follow-up.

The patient was advised to undergo a follow-up MRI to assess response one month after the start of the medication. At the one-month follow-up, the patient showed no improvement in his visual deficits and no new symptoms. MRI showed a progression of the lesion (33 mm x 35 mm x 33 mm) (Figure 2). Since the lesion was progressing rapidly despite medical treatment and visual symptoms remained unchanged, a surgical intervention aiming to get a pathological diagnosis and decompress the cavernous sinus was indicated. The patient underwent an endoscopic endonasal biopsy and cytoreductive surgery in the following days.

Pathology

Microscopic examination was consistent with adenocarcinoma of gastrointestinal origin, without any trace of pituitary adenoma. MIB-1 immunohistochemistry showed a high proliferative activity in the tumor. The specimen showed a very similar microscopic aspect to the primary esophageal adenocarcinoma, indicating metastasis. No sign of prolactinoma was seen on the final pathology.

Outcome and follow-up

**Figure 3:**
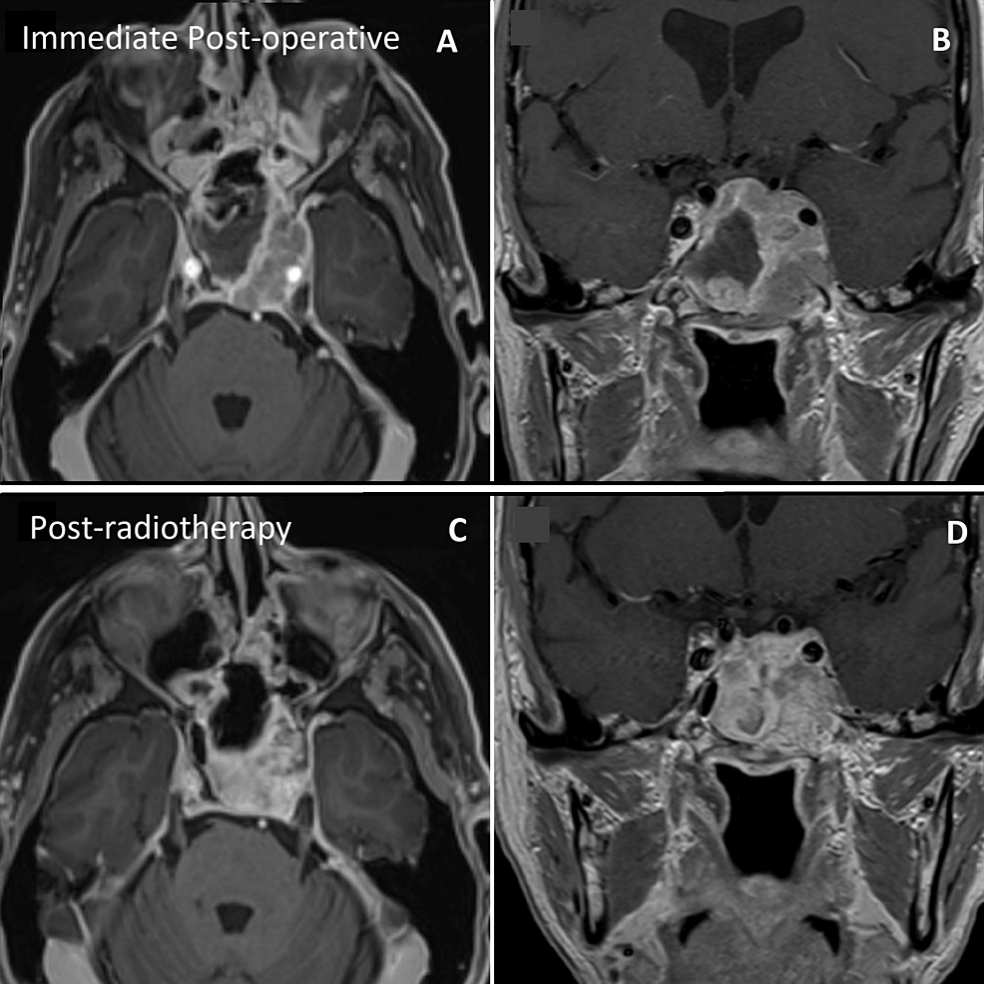
Postoperative and post-radiotherapy imaging A, B: Axial (A) and coronal (B) T1-weighted gadolinium-enhanced MRI showing the postoperative extent of the resection. Approximately 50% of the lesion was resected. C, D: Axial (C) and coronal (D) contrast-enhanced sequences showing the lesion’s partial response to the radiotherapy course MRI: magnetic resonance imaging

The patient had an uncomplicated postoperative course and was discharged home six days later. The third nerve palsy improved partially in the immediate postoperative period with improved eyelid opening and less marked diplopia. Postoperative MRI showed a 50% resection of the tumor, with residual tumor mainly in the cavernous sinus (Figure 3). In the context of the new metastasis, a pan-body PET scan was performed, which did not reveal any new metastatic lesion.

Following diagnosis, adjuvant treatment consisted of fractionated radiotherapy (20 Gy in five fractions). MRI two months after radiotherapy showed a 50-60% reduction of the residual tumor (Figure 3). At the last follow-up, four months after surgery, both his diplopia and ptosis kept on improving.

## Discussion

Composite tumors of the pituitary consisting of metastasis within an adenoma are extremely rare, with only 37 cases reported so far to our knowledge [6,7]. Of the 37 reported cases of metastasis within adenomas, 20 were found in non-functioning adenomas, seven in prolactinomas [8-14] (Table 2), four in adenomas that are GH-secreting, three secreting adrenocorticotropic hormone (ACTH), and three cases did not report the specific pathology of the adenoma [6]. The most common clinical presentation was vision loss in 21 cases. Six cases reported hormonal abnormalities (two Cushing’s syndrome, three acromegalies, and one hypopituitarism). The primary cancer of these metastasis involved lungs (13 cases), renal system (five cases), breast (six cases), colon (three cases), stomach (one case), prostate (one case), pancreas (one case), mediastinum (one case), melanoma (one case), and liver (one case). In three instances, no primary tumor could be identified. Of the 37 reported cases, 17 reported postoperative overall survival. It is no surprise that these patients fared worse than standard adenomas, with a median survival of 3.5 months and a mean survival of 4.7 months (0-24 months) following diagnosis [6]. Survival in these patients is often related to the prognosis of the primary tumor.

**Table 2:** Studies in the literature reporting composite tumors of metastasis in a prolactinoma

Study	Primary	Presentation	Age, years	Sex	Overall survival after surgery
Van Seters et al., 1985 [13]	Colon	Visual deficit	66	F	12 days
Abe et al., 1997 [8]	Mediastinum	Visual deficit	46	F	6 months
Hanna et al., 1999 [9]	Lung	Headache	42	F	Not reported
Komninos et al., 2004 [10]	Hepatocarcinoma	Visual deficit	68	M	4 months
Rotondo et al., 2013 [11]	Lung	Headache	66	M	Died before surgery
Thewjitcharoen et al., 2014 [12]	Colon	Visual deficit	65	M	9 months
Yang et al., 2017 [14]	Melanoma	Visual deficit	62	F	>22 months

In the seven previously reported cases of prolactinomas seeded by metastasis, none reported a spontaneous normalization of prolactin. This is most likely due to the fact that all patients received medical or surgical treatment upon presentation. The cases reported by Yang et al. [14] and Thewjitchroen et al. [12] both had surgical resection of the prolactinoma with no prior medical treatment. The initially elevated prolactin levels normalized postoperatively in these patients and pathology showed a composite tumor. Komninos et al. also reported normalization of prolactin (initially significantly elevated) within one week after surgery of the tumor [10]. In this case, however, pathology could only show metastatic tissue from a hepatic carcinoma. Hanna et al.'s case had initially high levels of prolactin; bromocriptine was initiated, which significantly lowered it. The patient, however, clinically deteriorated due to apoplexy and had surgery [9]. Abe et al. [8] and Van Seters et al. reported normalization of prolactin postoperatively [13]; both cases were already under medical treatment with bromocriptine. Abe et al. reported a case of a known prolactinoma, diagnosed and medically treated years prior, with elevated prolactin at the time of diagnosis. The patient sought consultation more than 10 years later; prolactin was almost within normal range, but MRI showed a significant lesion [8].

Differential diagnoses when prolactin levels spontaneously normalize in the setting of a suspected prolactinoma include necrosis of the functioning parts of the tumor, pituitary apoplexy, de-differentiation of the tumor, and destruction by another tumor. The aforementioned cases with prolactinoma composite tumors reported their concern about the fact that pre-treatment prolactin was not as high as they would have thought when correlating with the diameter of the tumor [10-12,14]. Small or moderate elevation of prolactin in the setting of a voluminous macroprolactinoma could be another sign of an erroneous diagnosis.

It has been shown that radiological features of sellar tumors often fail to differentiate between typical adenomas and pituitary metastasis [15]. The rapid growth of a usually benign and slowly growing prolactinoma, however, is not typical and should prompt investigations. Malignant transformation of a benign prolactinoma is the main differential diagnosis when such a clinical course occurs. The European Society of Endocrinology Clinical Practice guidelines for the management of aggressive pituitary tumors describe malignant transformation as a radiologically invasive tumor, unusually rapid tumor growth rate, or clinically relevant tumor growth despite optimal standard therapies (surgery, radiotherapy, and conventional medical treatments) [16]. Multiple studies have reported rapid growth in cases of malignant transformation of prolactinomas [17]. Some malignant prolactinomas were also reported to metastasize, which further complicates the diagnosis [17-20]. In cases of malignant prolactinomas, however, prolactin dosage tends to increase [16,17].

GI tract neoplasms have been identified as the primary neoplasm in four out of the eight prolactinoma composite tumor cases reported (including our case). This could suggest a certain tropism for GI tract primary tumors to seed into existing prolactinomas, although statistical analysis cannot validate this statement.

Our case showed a sellar and parasellar lesion with initial radiological and biochemical characteristics consistent with the diagnosis of a probable prolactinoma. The initially high prolactinemia, the slow growth over the course of the year, and the complete change in the pattern of growth years later increase the probability of a prolactinoma diagnosis. Stalk compression or other drugs usually do not increase prolactin levels as much as is seen in this case. Unfortunately, the patient did not receive medical treatment right away and was lost to follow-up, which would have further confirmed the diagnosis in the absence of pathological analysis. The behavior of the lesion became atypical at first with spontaneous prolactin normalization and even further when it grew substantially despite medical treatment. The hypothesized mechanism of prolactin normalization is that the more aggressive, invading metastasis invaded and destroyed the prolactin-secreting tissue of the prolactinoma. Komninos et al. [10] have reported a similar case, in which the patient presented with visual symptoms and elevated prolactin [438.6 ng/ml (normal level: <12.3 ng/ml)]. Prolactinoma was diagnosed. The patient had surgical resection before any medical treatment, and pathology revealed a complete invasion by metastasis from hepatocellular carcinoma, without any trace of prolactinoma.

A distinguishing feature of our case is the spontaneous normalization of prolactin levels without any treatment. This occurrence, especially in the presence of a known tumor elsewhere and rapid growth, should raise suspicion about the diagnosis of a composite tumor. Another interesting aspect is the time interval between the surgical “cure” of the primary tumor and metastatic recurrence into the prolactinoma, suggesting that even in the presence of a treated primary neoplasm, this diagnosis should be kept in mind. Due to the potential of prolactinomas to be metastatic sites, harboring an active primary neoplasm might be an argument in favor of surgical treatment of a prolactinoma instead of medical treatment to prevent metastasis, although it is a weak argument, as the pathology is extremely rare. A biopsy of the lesion is another option to consider when any atypical clinical or biochemical feature appears. However, given the rarity of this occurrence, the weight of this argument in surgical decision-making remains light. Any atypical tumor behavior, whether it is spontaneous normalization of prolactin, surprisingly low dosage of prolactin compared to the tumor volume, or rapid growth under medical treatment, should prompt physicians to consider a broader differential diagnosis and closer follow-up.

The main weakness of our report lies in the pathological analysis of the tumor: only metastasis was observed without any evidence of prolactinoma. However, we still believe the biochemical, clinical, and radiological courses are sufficient to point toward a composite tumor as the most likely diagnosis.

## Conclusions

Composite tumors consisting of a metastatic lesion in an adenoma are very rare entities. These lesions carry a higher morbidity and mortality burden and should be identified promptly. Normalization of prolactin prior to treatment, especially in a patient with a known primary neoplasm, should raise suspicion about metastasis. Prolactinoma growth despite medical treatment should also prompt the medical team to re-evaluate the working diagnosis and obtain a pathological sample.
